# Horror of three synergistic factors in THA: high mechanical stress, dissimilar metals, low elasticity stem: a case report

**DOI:** 10.1186/s42836-021-00091-7

**Published:** 2021-10-05

**Authors:** Seiya Ishii, Yasuhiro Homma, Takehisa Matsukawa, Tomonori Baba, Ayano Kubota, Kazuhito Yokoyama, Kazuo Kaneko, Muneaki Ishijima

**Affiliations:** 1 Department of Orthopaedic Surgery, Juntendo University, 2-1-1 Hongo, Bunkyo-ku, Tokyo, 113-0033 Japan; 2 Department of Epidemiology and Environmental Health, Juntendo University Faculty of Medicine, 2-1-1 Hongo, Bunkyo-ku, Tokyo, 113-8421 Japan; 3 Department of Forensic Medicine, Juntendo University Faculty of Medicine, 2-1-1 Hongo, Bunkyo-ku, Tokyo, 113-8421 Japan; 4 Department of Epidemiology and Social Medicine, International University of Health and Welfare Graduate School of Public Health, 4-1-26 Akasaka, Minato-ku, Tokyo, 107-8402 Japan

**Keywords:** Total hip arthroplasty, Large femoral head, Cobaltism, Corrosion

## Abstract

**Background:**

A large-diameter femoral head is effective in preventing dislocation after total hip arthroplasty. However, although rare, catastrophic stem tribocorrosion may occur at the head-stem junction.

**Case presentation:**

A 70-year-old woman underwent revision surgery 7.5 years after total hip arthroplasty because of catastrophic stem corrosion with dissociation of the metal head (cobalt/chromium) and stem (TiMo12Zr6Fe2). Abnormal levels of cobalt were found in the intra-articular fluid, capsule, hip muscle, and blood. Revision surgery was performed via the direct anterior approach. The well-fixed femoral stem was explanted, and a cemented stainless stem with stainless head was implanted. Three months after the revision surgery, the cobalt concentration in the blood had decreased to normal.

**Conclusions:**

Stem dissociation in the present case might have been caused by synergistic combination of a 36-mm-diameter femoral head and long neck length offset with high frictional torque, a cobalt-chromium head with a high risk of galvanic corrosion, and a TMZF (TiMo12Zr6Fe2) alloy stem with a low Young’s modulus of elasticity. The combination of these factors must be avoided.

## Background

Although total hip arthroplasty (THA) has excellent patient satisfaction and long-term survivorship, post-THA dislocation is one of its most serious complications [1]. Various attempts have been made to reduce the dislocation rate, including the surgical approach and determining the optimal implant positioning angle [2]. A large-diameter femoral head is another effective option because it increases the oscillation angle and jumping distance, thereby preventing postoperative dislocation [3, 4]. A randomized, controlled study revealed that THA with a 36-mm (instead of 28-mm) femoral head decreased postoperative dislocation [5]; however, despite its anti-dislocation effect, catastrophic stem tribocorrosion occurred at the head-stem junction (*i*.*e*., trunnionosis). The reported patient risk factors for trunnionosis are obesity, use of large metal heads, and long neck length [6].

We report a Japanese woman with abnormal cobalt levels in the intra-articular fluid, capsule, hip muscle, and blood who had early implant failure and required revision surgery 7.5 years after THA with a 36-mm metal head and TMZF (TiMo12Zr6Fe2) alloy stem. The patient provided consent for the publication of her case data.

## Case presentation

A 70-year-old woman (height 165 cm, weight 75 kg) with acute right hip pain was referred to us. She had undergone THA 7.5 years previously for hip osteoarthritis. The THA had been performed via the posterior approach using a 36-mm cobalt/chromium head and 5-mm neck (Stryker Inc., Tokyo, Japan), cementless stem (Accolade TMZF Hip Stem, 127°, #3; Stryker), cementless acetabular component (50 mm Trident Acetabular shell; Stryker), and X3 polyethylene insert. Up to 6 months prior to this admission, she could walk comfortably without a cane. She and others then started to hear abnormal squeaking sounds while she was walking.

**Fig. 1:**
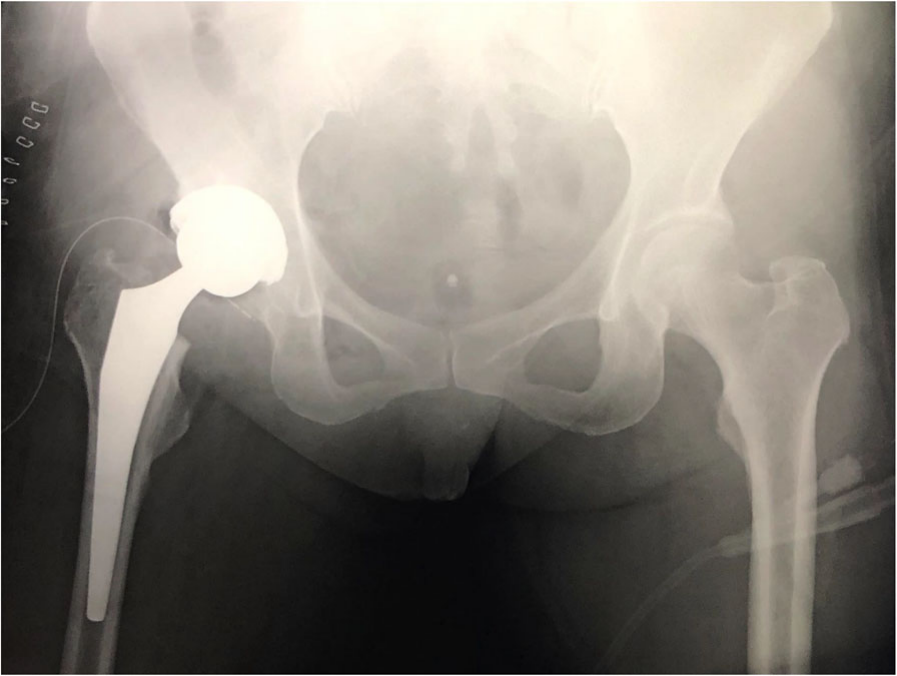
Anteroposterior plain radiography of both hips after total hip arthroplasty (THA)

**Fig. 2:**
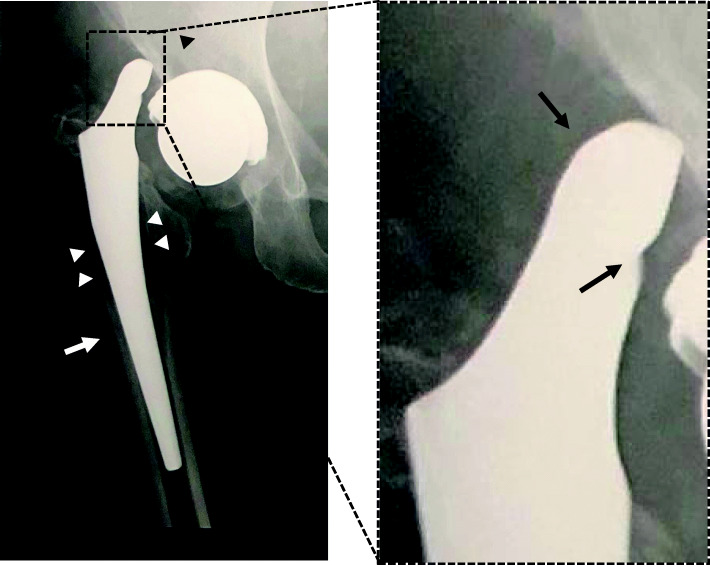
Radiography obtained 7.5 years after total hip arthroplasty (THA). There is dissociation of the femoral head from the head-stem trunnion (right panel, box) after THA (right panel, black arrows), radiolucency (left panel: black arrowhead, and white arrowheads), and cortical hypertrophy (white arrow)

Laboratory data showed a normal C-reactive protein level (0.12 mg/dl) but abnormal serum levels of cobalt (7.0 μg/L, normal < 1 μg /L) and chromium (3.7 μg/L, normal < 5 μg /L) [7]. Radiography performed just after the previous THA and prior to this admission showed no abnormalities in cup inclination (33°) or anteversion angle (19°) (Fig. 1). However, each offset in the right hip was longer than that in the left hip (femoral/acetabular/total offset: 43.7/38.6/82.3 (right) and 42.1/33.1/75.2 (left)). Leg length was 2 mm longer on the operative side than on the healthy side [8]. Plain radiography at the time of the present admission showed implant dissociation between the stem and head, with stem tip malformation (Fig. 2). Plain computed tomography and magnetic resonance imaging showed a mass (72 × 22 × 32 mm) that was partially calcified around the hip joint. We therefore suspected femoral head-stem trunnion dissociation secondary to corrosion after THA with a large-diameter metal head that required revision surgery.

**Fig. 3:**
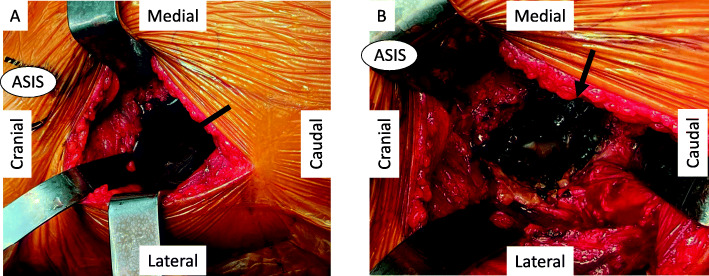
Intraoperative photographs. **A** Pigmented metal around the joint (arrow). **B** Black granulation tissue adhering to the inner surface of the joint capsule (arrow)

Surgery was performed using a direct anterior approach on a traction table [9]. Before opening the capsule, the rectus femoris and tensor fascia lata were biopsied for metal ion testing. Surgical findings included a joint capsule filled with black fluid and black granulation tissue (Fig. 3A, B), as well as thickened fibrous tissue. The anterior capsule was sent for pathological examination. Tissues debrided around the capsule were severely affected by metal ions. The pigmented granulation tissue in the joint was removed.

**Fig. 4:**
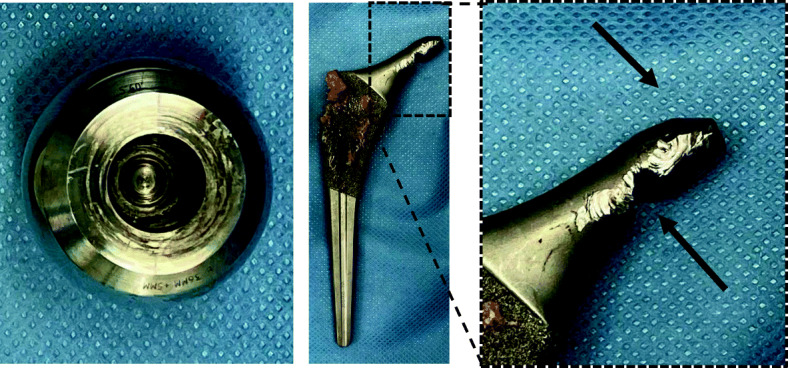
Photographs of the extracted instrumentation. Left panel: metal debris inside the metal head. Middle and right panels: significant deformation at the head-stem trunnion (arrows)

**Fig. 5:**
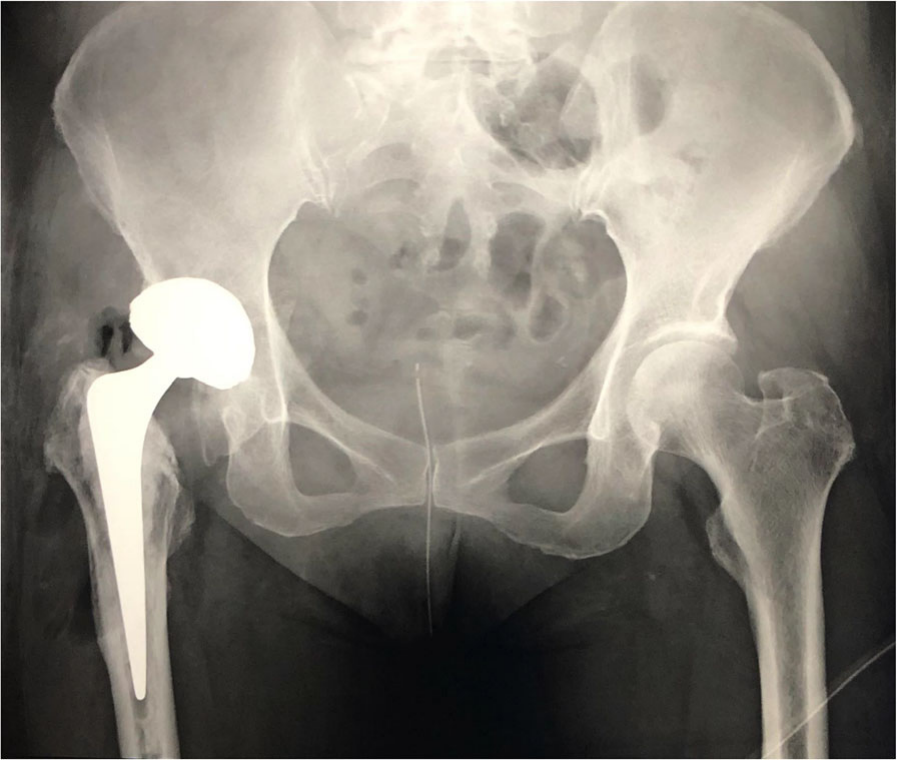
Postoperative radiography showing the cemented femoral stem

There was no loosening of the cup or stem. The removed head-stem junction was severely deformed and worn (Fig. 4). The acetabular cup was well fixed. The polyethylene liner was replaced with a metal liner for the dual mobility cup. The well-fixed femoral stem was explanted without fracture, and a cemented stem was implanted [Exeter V40 (offset 37.5 mm, # 2); Stryker] (Fig. 5). Total right hip femoral offset was intentionally decreased from 82.3 to 63.8 mm (lower than that of the left hip). Leg-length discrepancy was corrected to within 1 mm.

**Fig. 6:**
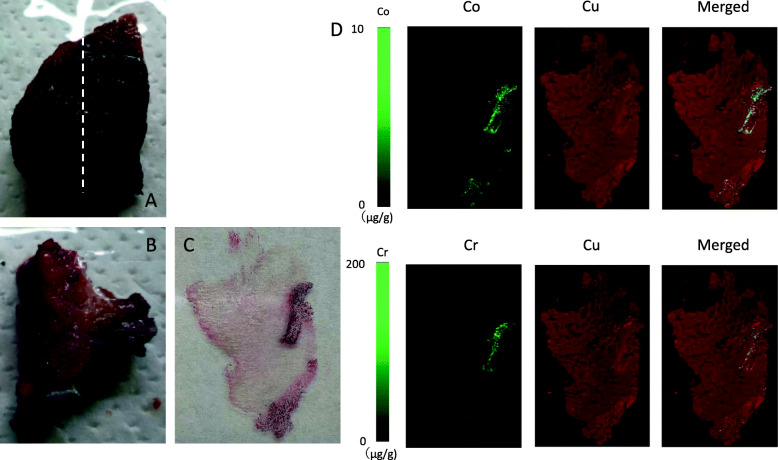
Photographs of extracted specimens and distribution of cobalt or chromium in the joint capsule. **A** Anterior capsule. White dotted line shows the slice levels in panels B–D. **B** Slice of the anterior capsule. **C** Serial section from tissue in **B** (hematoxylin-eosin, × 40). **D** Semi-quantitative laser ablation-inductively coupled plasma mass spectrometry images. Left panels: quantitative images of cobalt or chromium. Middle panels: images of copper distribution inside the joint capsule as a position reference. Right panels: merged images of cobalt or chromium and copper distributions

The microbial cultures of the joint fluid were negative. Pathological examination revealed necrotic tissue with fibrosis. Cobalt/chromium metal levels were elevated in synovial fluid, joint capsule, rectus femoris, and fascia lata (Table 1). Frozen tissue sections (5-μm thick) of the anterior joint capsule were processed. The distribution of cobalt or chromium in the joint capsule—determined by laser ablation (NWR213; Elemental Scientific Lasers, Tokyo, Japan) and inductively coupled mass spectrometry (LA-ICP-MS) (Agilent 8800; Agilent Scientific, Santa Clara, CA, USA)—is shown in Fig. 6. Concentrations were estimated using the results of conventional LA-ICP-MS after acid digestion of the neighboring tissue. Both cobalt and chromium were distributed on the inner surface of the joint capsule as small particles. Semiquantitative LA-ICP-MS images showed cobalt/chromium on the inner surface of the joint capsule (Fig. 6D).

**Table 1:**
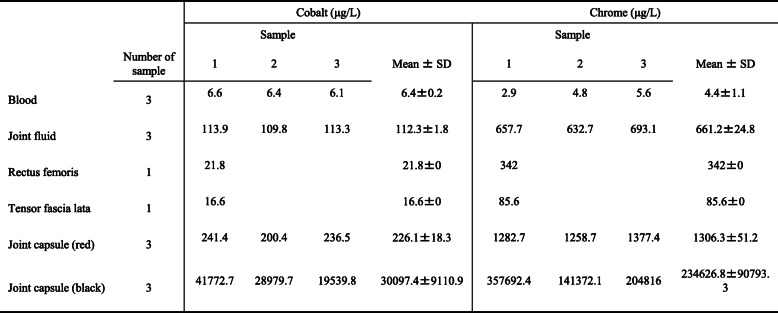
Distribution of cobalt and chromium ions in each living tissue biopsied in the reported patient after implant failure

**Fig. 7:**
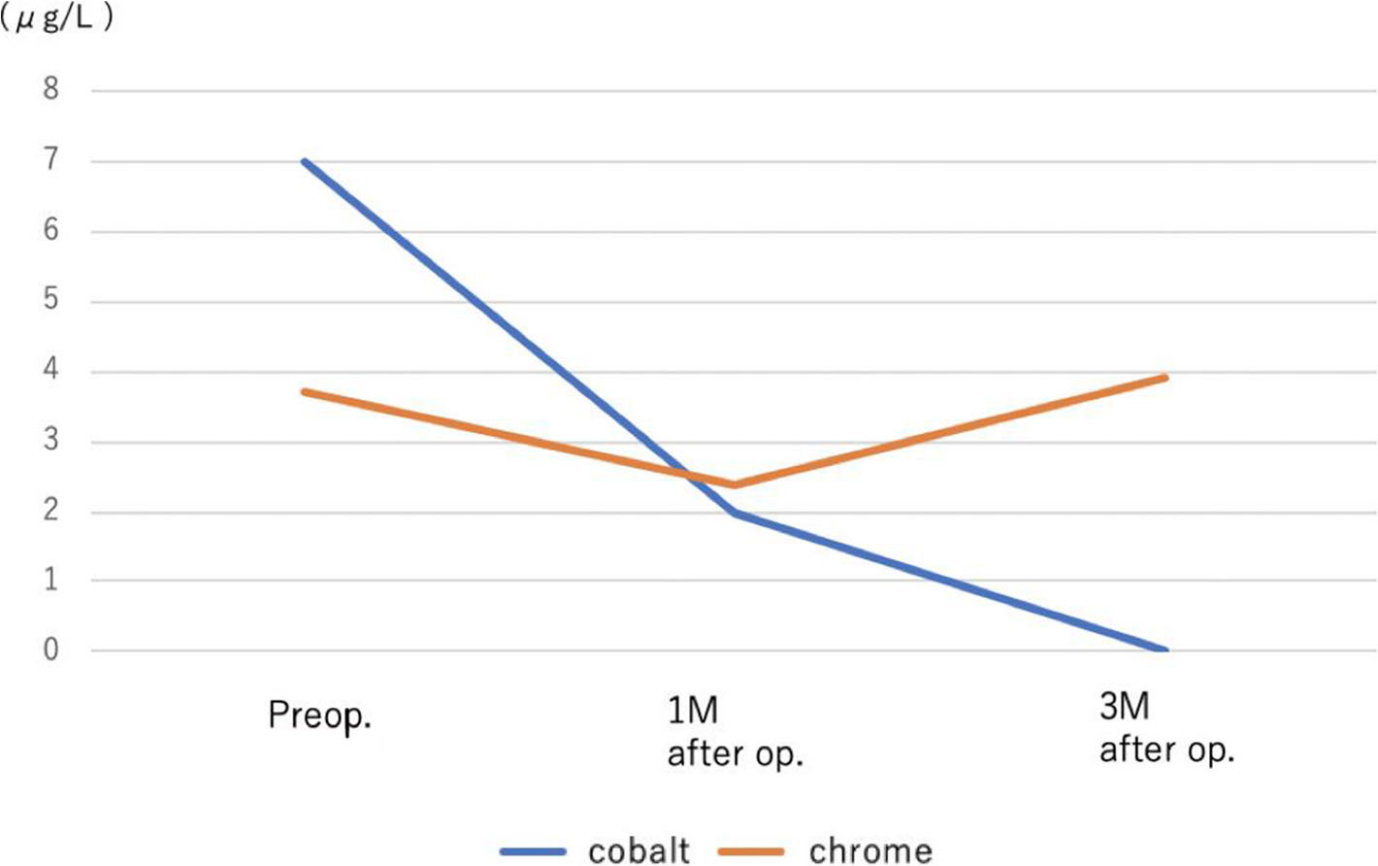
Changes in postoperative blood metal (cobalt and chromium) concentrations. M, month(s); Preop., preoperatively; op., operation

Immediate alleviation of the patient’s pain was observed postoperatively, and painless walking was possible 1 month later. Her blood cobalt concentrations decreased with time and were undetectable at 3 months postoperatively (Fig. 7).

## Discussion

We experienced a patient who had undergone THA with a 36-mm metal head and developed catastrophic trunnionosis with elevated blood cobalt levels that required revision surgery 7.5 years later. We concluded that the use of a 36-mm cobalt/chromium head with a dissimilar metal stem should be severely restricted.

Although previous reports suggested other risk factors—*e*.*g*., body weight, high neck offset, and activity level [10–18]—we believe that the three major potentially linked factors that contributed to the onset of trunnionosis in our patient were increased mechanical stress at the trunnion, contact of dissimilar metals at the trunnion, and use of an Accolade TMZF stem.

First, we think that the increased mechanical stress at the trunnion (*i*.*e*., fretting corrosion) played a major role in the development of trunnionosis. Increased mechanical stress is induced by multiple factors (*e*.*g*., greater body weight, increased activity level, longer and higher neck offset, large head size), and it is speculated that these factors synergistically generate high mechanical stress at the trunnion [10–12, 19]. In our case, the patient’s body weight, high neck length, longer femoral offset compared with the left hip, and large femoral head synergistically increased the mechanical stress.

Although it is difficult to determine a body mass index (BMI) cutoff value, it is obvious that heavier patients have a greater risk of increased mechanical stress at the trunnion. Previous studies have reported that the risk of trunnionosis is high in patients with a BMI > 30 kg/m^2^ [6], particularly men with high BMI (91.4% of trunnionosis cases occur in men) [11]. Because our patient was a woman weighing 75 kg with a BMI of 27.5 kg/m^2^ and no increased activity level (although the BMI was only just acceptable), the trunnionosis was likely due to other factors leading to unacceptable mechanical stress.

Although it is unclear which femoral head diameter is safest to use to avoid trunnionosis, a diameter of ≥36 mm seems to put patients at higher risk. However, trunnionosis may occur with a 32-mm femoral head if other risk factors for increased mechanical stress are present (*e*.*g*., heavy body weight, large neck offset). Dan *et al* reported a case of trunnionosis with a 32-mm femoral head in a patient weighing 90 kg [14]. Considering these points, surgeons should avoid unnecessary leg lengthening, an inappropriate femoral offset, and the use of a large femoral head.

Second, contact between dissimilar metals can cause galvanic corrosion due to an electrochemical process resulting from electron flow [15, 20]. Although titanium stems and cobalt/chromium heads have individual advantages, the combination of these metals could potentially cause a synergistic effect causing corrosion, especially under great mechanical stress. The implant should be selected after considering the risks and benefits of these variables.

Third, the nature of the Accolade TMZF negatively affects the onset of trunnionosis. Stems made of TMZF alloy have a low Young’s modulus of elasticity. The Young’s modulus is proportional to the flexural rigidity and that of Ti-6Al-4 V is 119 GPa while that of TMZF is 82 GPa [16]. The low flexural rigidity of TMZF is a risk factor for taper corrosion [17]. Krishnan *et al* reviewed modular femoral stems and found the most failures with a TMZF stem and cobalt/chromium stem combination [18]. Thus, combined ABG2 and Rejuvenate modular stems (Stryker, Kalamazoo, MI, USA) were recalled because of high revision rates due to corrosion. In addition, a systematic review reported that the Accolade TMZF poses a serious risk of trunnionosis, with trunnionosis occurring in 34/46 cases using the Accolade TMZF [11]. Although low flexural rigidity may have the advantage of normal load transmission to native bone, appropriate strength with high safety should be guaranteed.

Elevated cobalt/chromium levels are another important issue. According to Dundon *et al*, our patient had enough cobalt/chromium to cause multiple systemic complications, and revision surgery would be recommended even without implant failure [12]. Sometimes reported as “cobaltism,” high levels of blood cobalt cause systemic complications, including vision loss, hearing loss, cognitive decline, hypothyroidism, lymphopenia, and fatal cardiomyopathy [21–25]. Our patient had lower cobalt/chromium levels than reported by Ikeda *et al* [25], possibly because (1) the dissociation occurred before the metal concentration increased, implying the importance of early detection of trunnionosis, and/or (2) although the trunnion was massively deformed, there was minimal deformation of the metal femoral head.

Judging from the anterior capsule images of the metal concentration in the black intra-articular fluid and anterior capsule, the metal first accumulates at the joint, with the capsule a possible barrier to expansion around the hip joint. Although the cobalt blood level decreased to the normal range within 3 months after the revision surgery, metal ions were detected in the hip muscle at the time of the surgery, where there was no obvious contamination. As cobalt is thought to accumulate in strained muscle, careful and continued observation of this patient is necessary.

## Conclusion

The present patient had abnormal levels of cobalt/chromium in intra-articular fluid, capsule, hip muscle, and blood, and underwent revision surgery 7.5 years after the initial THA due to catastrophic stem corrosion at the trunnion. Use of a 36-mm metal femoral head combined with a low elasticity stem must be severely restricted. Overall, the present case suggests that the systematic effects of metal should be investigated.

## Data Availability

All data generated or analyzed during this study are included in this published article.

## Abbreviations

THA: Total hip arthroplasty
BMI: Body mass index
